# A Perfect Storm: Increased Colonization and Failure of Vaccination Leads to Severe Secondary Bacterial Infection in Influenza Virus-Infected Obese Mice

**DOI:** 10.1128/mBio.00889-17

**Published:** 2017-09-19

**Authors:** Erik A. Karlsson, Victoria A. Meliopoulos, Nicholas C. van de Velde, Lee-Ann van de Velde, Beth Mann, Geli Gao, Jason Rosch, Elaine Tuomanen, Jon McCullers, Peter Vogel, Stacey Schultz-Cherry

**Affiliations:** a Department of Infectious Diseases, St. Jude Children’s Research Hospital, Memphis, Tennessee, USA; b Department of Immunology, St. Jude Children’s Research Hospital, Memphis, Tennessee, USA; c Department of Pediatrics, University of Tennessee Health Science Center, Memphis, Tennessee, USA; d Veterinary Pathology Core, St. Jude Children’s Research Hospital, Memphis, Tennessee, USA; University of Pittsburgh School of Medicine

**Keywords:** *Streptococcus pneumoniae*, coinfection, influenza, lung infection, obesity

## Abstract

Obesity is a risk factor for developing severe disease following influenza virus infection; however, the comorbidity of obesity and secondary bacterial infection, a serious complication of influenza virus infections, is unknown. To fill this gap in knowledge, lean and obese C57BL/6 mice were infected with a nonlethal dose of influenza virus followed by a nonlethal dose of *Streptococcus pneumoniae*. Strikingly, not only did significantly enhanced death occur in obese coinfected mice compared to lean controls, but also high mortality was seen irrespective of influenza virus strain, bacterial strain, or timing of coinfection. This result was unexpected, given that most influenza virus strains, especially seasonal human A and B viruses, are nonlethal in this model. Both viral and bacterial titers were increased in the upper respiratory tract and lungs of obese animals as early as days 1 and 2 post-bacterial infection, leading to a significant decrease in lung function. This increased bacterial load correlated with extensive cellular damage and upregulation of platelet-activating factor receptor, a host receptor central to pneumococcal invasion. Importantly, while vaccination of obese mice against either influenza virus or pneumococcus failed to confer protection, antibiotic treatment was able to resolve secondary bacterial infection-associated mortality. Overall, secondary bacterial pneumonia could be a widespread, unaddressed public health problem in an increasingly obese population.

## IMPORTANCE

Worldwide obesity rates have continued to increase. Obesity is associated with increased severity of influenza virus infection; however, very little is known about respiratory coinfections in this expanding, high-risk population. Our studies utilized a coinfection model to show that obesity increases mortality from secondary bacterial infection following influenza virus challenge through a “perfect storm” of host factors that lead to excessive viral and bacterial outgrowth. In addition, we found that vaccination of obese mice against either virus or bacteria failed to confer protection against coinfection, but antibiotic treatment did alleviate mortality. Combined, these results represent an understudied and imminent public health concern in a weighty portion of the global population.

## INTRODUCTION

Epidemiological and laboratory studies have shown obesity to be a risk factor for increased severity and mortality from influenza virus infection. Since the 2009 pandemic, obese individuals are now considered a high-risk group for developing severe disease during seasonal and pandemic influenza outbreaks (1–4). While the exact mechanisms are yet to be elucidated, impaired immune functionality and increased wounding to the lung epithelium have been implicated in the increased disease severity (1, 4, 5). Secondary bacterial coinfection has been recognized as a significant complication to influenza severity (6). Given the continual, global expansion in the obese population (7) and increased susceptibility of the obese host to infectious disease (8–10), it is imperative gain a better understanding of these mechanisms and risk factors.

A substantial proportion of the mortality observed during both seasonal and pandemic influenza outbreaks is attributed to bacteria-associated pneumonia, especially from *Streptococcus pneumoniae* (11). Increased efficiency of bacterial colonization following an influenza virus infection contributes to destruction of the lung and airway epithelium, which exposes bacterial binding sites, disrupts mechanical clearance mechanisms, increases inflammation, enhances availability of bacterial nutrient substrates, and alters the host’s ability to mount specific immune responses (6, 12). Interestingly, influenza virus infection alone in the obese host has been associated with all of these risk factors, including increased airway damage, altered inflammation, and decreased immune function (1, 13). These factors raise the question of synergistic destruction between secondary bacterial coinfection in the setting of obesity; however, the mechanism of copathogenesis of obesity and secondary bacterial coinfection is unknown. Therefore, we sought to fill this gap in knowledge and investigate secondary bacterial infection in obese mice by using an established coinfection model (14).

Both diet-induced and genetically obese C57BL/6 mice demonstrated significantly enhanced morbidity and mortality compared to lean controls, irrespective of bacterial or influenza virus strain or timing of bacterial administration post-influenza virus infection. Increased distribution throughout the lung of virus and bacteria after secondary challenge, aberrant immune responses, and additive obesity- and influenza-driven increases in expression of bacterial receptors appeared to raise the severity of coinfection. Importantly, vaccinating obese mice against either influenza virus or pneumococcus failed to protect against the increased morbidity associated with coinfection; however, antibiotic treatment succeeded in protecting obese mice from secondary bacteria-associated mortality. Overall, these results indicate that secondary bacterial pneumonia is likely to be a problem in an increasingly obese population.

## RESULTS

### Secondary bacterial infection leads to increased mortality in obese mice regardless of viral strain or timing of bacterial infection.

**FIG 1:**
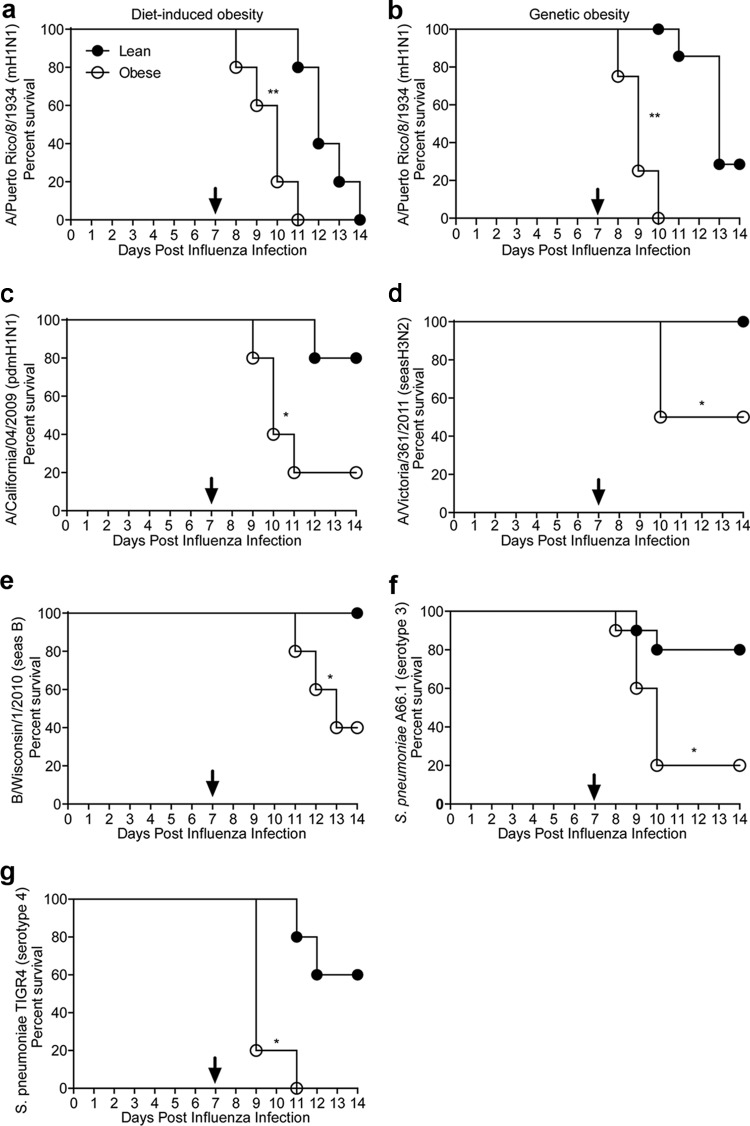
Obesity increases mortality associated with secondary bacterial infection regardless of viral or bacterial strain. (a and b) Diet-induced (a) or genetically obese (b) mice and lean controls were infected with influenza virus A/Puerto Rico/8/1934 and then challenged with *Staphylococcus pneumoniae* strain D39x (serotype 2) at 7 days postinfection. (c to f) Similarly, genetically obese and lean controls mice were challenged with influenza virus A/California/04/2009 (human pandemic H1N1) (c), A/Victoria/361/2011 (human seasonal H3N2) (d), or B/Wisconsin/1/2010 (human seasonal B) (e) and then challenged with bacteria 7 days post-influenza virus infection. (f and g) Finally, genetically obese mice and lean controls were infected with influenza virus A/California/04/2009 and then challenged with *S. pneumoniae* strain A66.1 (serotype 3) (f) or TIGR4 (serotype 4) (g) at 7 days postinfection. Mortality was monitored for 7 days postbacterial challenge. Survival was analyzed by a log rank (Mantel-Cox) test. Data are representative of at least two separate experiments, with 5 mice per group. *, *P* < 0.05. Arrows indicate the day of bacterial challenge.

Diet-induced (DIO) and genetically obese (B6.Cg-Lep*ob*/J; *ob*/*ob*) mice, along with respective lean or wild-type (C57BL/6; WT) controls were challenged with a sublethal dose of influenza A/Puerto Rico/8/1934 (PR8) virus, followed by a sublethal dose of *S. pneumoniae* strain D39x (serotype 2) 7 days later. Singular infections caused no morbidity or mortality (see Fig. S1a and b in the supplemental material). As expected, lean and WT mice succumbed to coinfection approximately 5 to 7 days post-bacterial inoculation (6, 14). Interestingly, DIO (Fig. 1a) and genetically obese (Fig. 1b) mice succumbed to secondary bacterial infection much earlier, beginning 24 h post-bacterial challenge, with 100% mortality by 72 h (Fig. 1). Since both DIO and *ob*/*ob* mice showed increased susceptibility to secondary bacterial infection, further studies were conducted with *ob*/*ob* mice as a model for morbid obesity, based on their numerous metabolic factors, including hyperglycemian (64).

10.1128/mBio.00889-17.1 FIG S1  Individual infections are nonlethal in both lean and obese mice. Lean (solid) and obese (open) mice were infected with low doses of (a) influenza virus A/California/04/2009 or (b) *S. pneumoniae* strain D39x. Weight loss was monitored for 7 days postinfection. *n* = 5 mice/group. Viral and bacterial titers were measured in the lungs as well as nasal lavages at days 7, 8, and 9 for influenza virus infection and days 1, 2, and 3 for post-bacterial infection. *n* = 3 mice/group/time point. *, *P* < 0.05. Download FIG S1, TIF file, 2.8 MB. Copyright © 2017 Karlsson et al. 2017 Karlsson et al. This content is distributed under the terms of the Creative Commons Attribution 4.0 International license.

Only certain influenza virus strains exacerbate secondary bacterial pneumonia in lean mice (15). To determine if this was true in obese animals, obese mice were inoculated with sublethal doses of influenza virus A/California/04/09 (CA/09 pdm H1N1) (Fig. 1c), A/Victoria/36/2011 (seasonal H3N2) (Fig. 1d), B/Wisconsin/1/2010 (B) (Fig. 1e), A/Brisbane/59/2007 (seasonal H1N1) (Fig. S2a), or A/Indiana/06/2011 (variant vH3N2) (Fig. S2b) followed by a sublethal dose of *S. pneumoniae* D39x 7 days later. All obese mice exhibited increased mortality compared to lean mice, with increased mortality ranging from 20% with the older seasonal H1N1 and vH3N2 viruses to 80% with pandemic H1N1 virus. This was not limited to the specific influenza virus strain. Increased disease severity was also seen with different pneumococcal serotypes. Inoculating obese mice with a sublethal dose of *S. pneumoniae* strain A66.1 (serotype 3), which is considered a low-pathogenicity strain (16, 17), at 7 days post-influenza virus inoculation resulted in 80% mortality, versus only 20% in control, lean mice (Fig. 1f). Similarly, challenging mice with a sublethal dose of *S. pneumoniae* strain TIGR4 (serotype 4), a highly invasive serotype (18), resulted in 100% mortality in obese mice versus 40% in control mice (Fig. 1g). All subsequent studies were performed with CA/09 pdmH1N1 virus.

10.1128/mBio.00889-17.2 FIG S2  Obesity increases mortality associated with secondary bacterial infection regardless of viral strain. Obese (open) and lean (solid) mice were challenged with influenza virus A/Brisbane/59/2007 (human seasonal H1N1) (a) or A/Indiana/08/2011 (variant H3N2) (b) and then challenged with bacteria 7 days post-influenza virus infection. Mortality was monitored for 7 days post-bacterial challenge. Survival was analyzed by a log-rank (Mantel-Cox) test. Data are representative of at least two separate experiments with 5 mice per group. *, *P* < 0.05. Download FIG S2, TIF file, 0.3 MB. Copyright © 2017 Karlsson et al. 2017 Karlsson et al. This content is distributed under the terms of the Creative Commons Attribution 4.0 International license.

The timing of bacterial challenge following influenza virus infection can significantly impact development of pneumonia in lean mice (19, 20). However, this is not true in obese animals. As expected, when lean mice were inoculated with bacteria 3, 7, or 10 days post-CA/09 pdmH1N1 virus infection, significant mortality was only observed at days 7 and 10 post-influenza infection. In contrast, obese mice had significant morality at all times, and the time to death decreased from ∼5.6 days in animals administered bacteria 3 days post-influenza virus infection to ∼1.6 days in those receiving bacteria at 10 days post-influenza virus infection (Fig. S3). Overall, these studies demonstrated that obese mice, regardless of the type of obesity, are more likely to succumb to a secondary bacterial challenge with *S. pneumoniae* after influenza. Intriguingly, the increased mortality is not limited to either a particular influenza virus or pneumococcal strain nor to the timing post-influenza virus infection, unlike responses in lean mice.

10.1128/mBio.00889-17.3 FIG S3  Timing of bacterial challenge does not decrease mortality associated with secondary bacterial infection in obese mice. (a) Lean (solid symbols) and obese (open symbols) mice were infected with influenza virus A/California/04/2009 and then challenged with *S. pneumoniae* strain D39x at day 3 (circle), day 7 (square), or day 10 (diamond) post-influenza virus infection. Mortality was monitored out to 14 days post-influenza virus infeciton. Survival was analyzed by a log-rank (Mantel-Cox) test. Data are representative of at least two separate experiments with 5 mice per group. *, *P* < 0.05. (b) Time to death was calculated based on the number of days to mortality after bacterial challenge. *, *P* < 0.05. Download FIG S3, TIF file, 0.6 MB. Copyright © 2017 Karlsson et al. 2017 Karlsson et al. This content is distributed under the terms of the Creative Commons Attribution 4.0 International license.

### Increased viral and bacterial levels and spread in obese mice.

**FIG 2:**
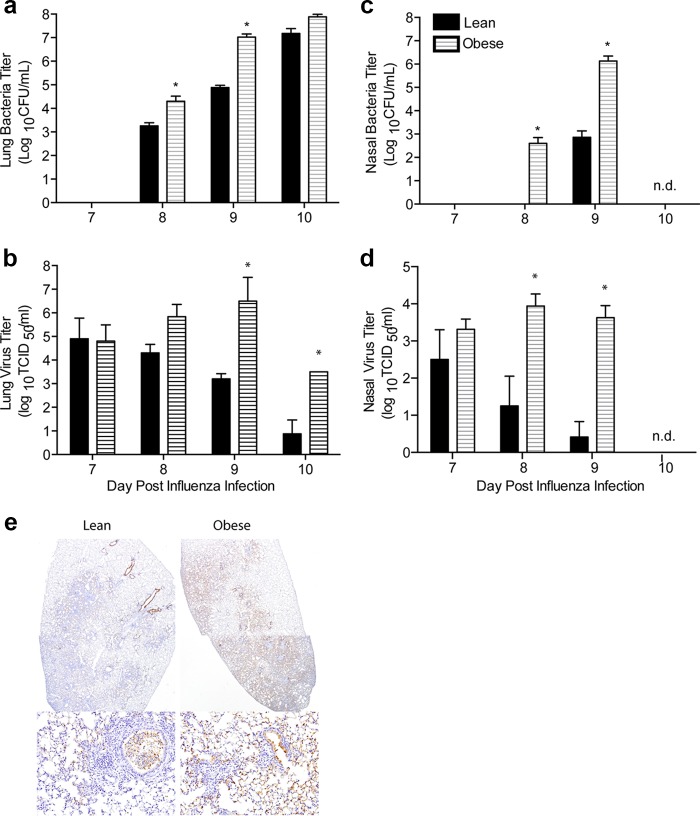
Obese mice have increased viral and bacterial titers following coinfection. Lean and obese mice were infected with influenza virus A/California/04/2009 and then challenged with bacteria 7 days post-influenza virus infeciton. Bacterial and viral titers were measured in the lungs (a and c) as well as in nasal lavage samples (b and d) prior to and every 24 h following bacterial coinfection. Data are representative of three separate experiments with 3 to 5 mice/group/time point. *, *P* < 0.05. (e) Lungs were obtained from perfused lean and obese mice 2 days post-bacterial coinfection (9 days post-influenza virus infection) and sectioned. Lung sections were stained against nucleoprotein of influenza virus. Sections are representative of two separate experiments with 3 mice/group.

**FIG 3:**
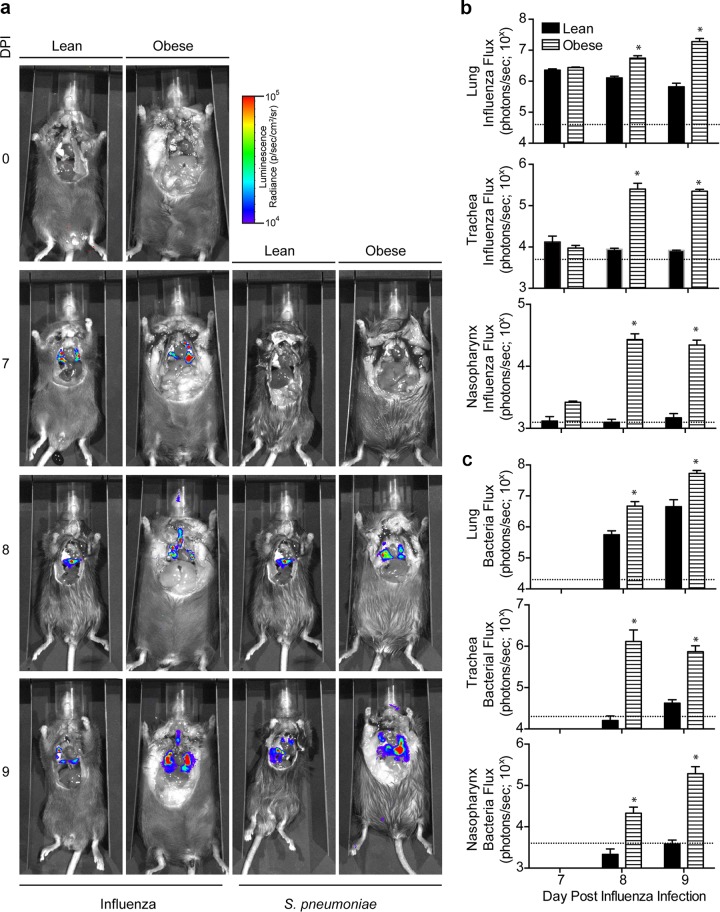
Bioluminescent imaging of virus and bacteria prior to and following coinfection confirmed increased spread in obese animals. Lean and obese mice were infected with influenza virus A/California/04/2009-NLuc and then challenged with bioluminescent D39x bacteria 7 days post-influenza virus infection. (a) Whole-animal viral and bacterial bioluminescence levels were measured in uninfected mice at day 7 post-influenza virus infection and every 24-h postcoinfection. (b and c) Luminescent flux was directly assessed for influenza virus (b) and bacteria (c) in the lung, trachea, and nasopharynx of individual animals. Images are representative of all animals in each group, and luminescent flu data are from 3 animals/group/time point. *, *P* < 0.05. Dotted lines indicate background levels in infected controls.

We next questioned whether this increased susceptibility was due to increased viral or bacterial load. At 1 day post-bacterial inoculation, there was a 1-log increase in lung (Fig. 2a) and nasal (Fig. 2b) viral titers in obese animals that increased to an ∼3-log increase by 2 days post-bacterial inoculation. In contrast, lean mice cleared the virus during the bacterial infection, whereas titers in the obese animals remained elevated until the time of death. Similarly, lung bacterial loads were greater than 1-log higher in the obese mice 1 day post-bacteria administration, increasing to 2 to 3 logs greater by 2 days postinoculation (Fig. 2c). This was even more evident in the upper respiratory tract, where bacterial titers reached >10^6^ by 2 days postinfection in obese animals (Fig. 2d). This was coupled with increased spread throughout the respiratory tract of obese animals, as demonstrated by immunohistochemical staining for the viral nucleoprotein (NP) in the lungs (Fig. 2e) and through the imaging of bioluminescent D39x bacteria (21) and pdmH1N1 virus (22) (Fig. 3). Both bacteria and virus were detected in the nasopharynx and trachea of obese mice, starting 1 day post-bacterial inoculation (Fig. 3a). Quantitation of the bioluminescent bacteria and virus flux in the nasopharynx, trachea, and lungs clearly showed increased spread in obese mice (Fig. 3b). All of these results are in contrast to individual infections in both lean and obese mice, which show viral clearance and decreased bacterial loads (Fig. S1 and S4). Overall, these data demonstrate that obese mice exhibit greater bacterial and viral loads that spread more extensively throughout the respiratory tract compared to lean mice.

10.1128/mBio.00889-17.4 FIG S4  Bioluminescent imaging of virus and bacteria following primary infections in lean and obese animals. Lean and obese mice were infected with influenza virus A/California/04/2009-NLuc or bioluminescent D39x bacteria. Whole-animal viral and bacterial bioluminescence levels were measured in uninfected mice at days 7, 8, and 9 post-influenza virus infection and at days 1, 2, and 3 post-bacterial infection. Download FIG S4, TIF file, 0.1 MB. Copyright © 2017 Karlsson et al. 2017 Karlsson et al. This content is distributed under the terms of the Creative Commons Attribution 4.0 International license.

### Numerous mechanisms for increased bacterial colonization in obese mice.

**FIG 4:**
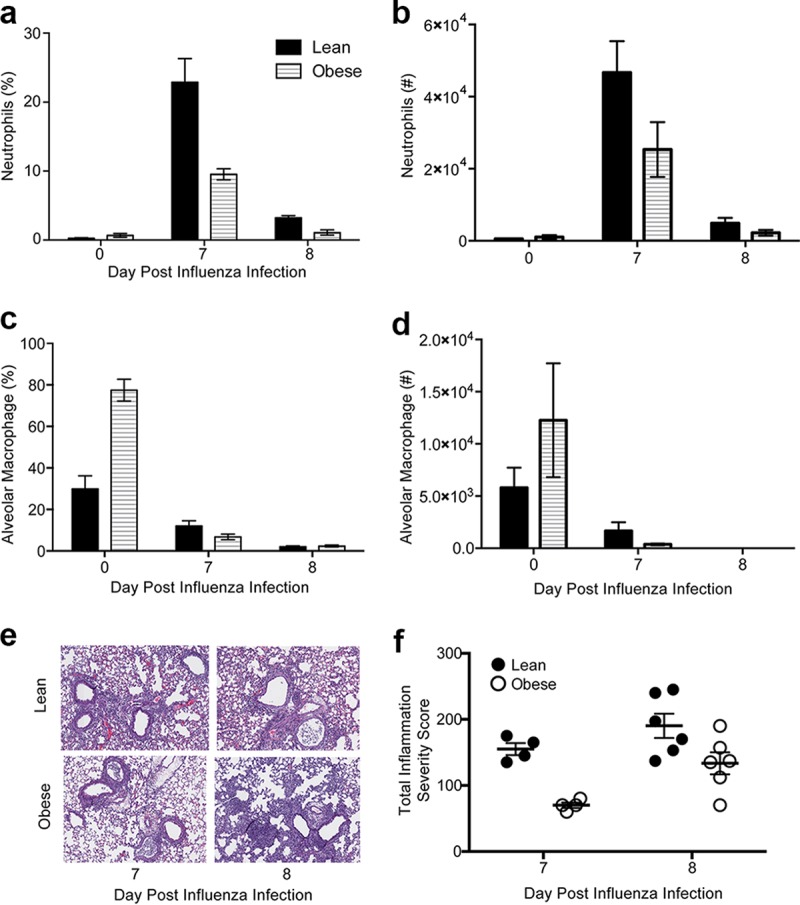
Obesity is associated with altered innate immune cells and deceased inflammation compared to lean controls. (a to d) Neutrophils (a and b) and alveolar macrophage populations (c and d) were assessed in bronchoalveolar lavage fluid from lean and obese mice prior to and following secondary bacterial challenge. Data are representative of two separate experiments with 3 to 5 mice/group/time point. *, *P* < 0.05. (e and f) Lungs were obtained from perfused lean and obese mice 7 days post-influenza virus challenge and 2 days post-bacterial coinfection (9 days post-influenza virus infection) and sectioned. Lung sections were stained with hematoxylin and eosin to determine overall inflammation (e) and blindly scored for overall inflammation (f). Sections are representative of two separate experiments with 3 mice/group.

Several mechanisms have been proposed for how influenza virus enhances subsequent bacterial infection from aberrant innate and adaptive immune responses (23–25) to increased binding sites and bacterial nutrients (12, 26–30). Obese mice had significantly lower percentages of lung neutrophils and alveolar macrophages (AM) at 7 days post-influenza virus infection (Fig. 4a and b), which further decreased after bacterial challenge. Neutrophil numbers quickly decreased in both lean and obese mice; however, lean animals still had significantly increased neutrophils (percentages) than obese animals (Fig. 4a and b). The decrease in AMs was even more striking, given that obese mice had significantly increased AMs prior to influenza virus infection (3 times higher), followed by a more substantial decrease 7 days post-influenza virus infection (Fig. 4c and d). This was also evident in the decreased overall inflammation in the lungs (interstitial and alveolar inflammation, alveolar protein, septal thickening hyaline membranes) of obese mice, similar to findings reported previously (1) (Fig. 4c and d).

**FIG 5:**
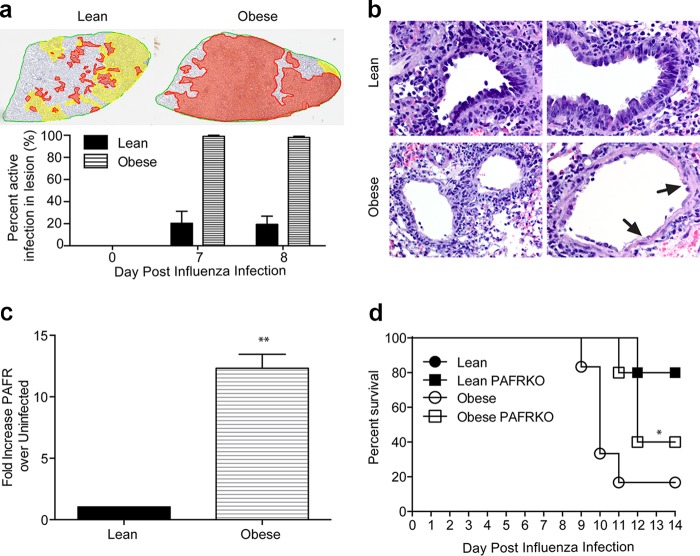
Increased exposure of PAFR expression in the lungs of obese mice. (a) Lungs were obtained from perfused lean and obese mice prior to infection, 7 days post-influenza virus challenge and 24 h post-bacterial coinfection (8 days post-influenza virus infection) and sectioned. Sections were stained for influenza virus NP, and “active” infection was quantified based on the intensity of staining. Data are representative of two separate experiments with 3 mice/group/time point. *, *P* < 0.05. (b) Obese mice have increased exposure of basement membrane (arrows) at day 7 post-influenza virus infection. (c) The fold increase in expression of PFAR (versus expression in uninfected animals) was quantified by Western blotting from the lungs of lean and obese mice prior to and 7 days post-influenza virus infection. Data are representative of two separate experiments with 3 mice/group/time point. *, *P* < 0.05. (D) Wild-type and PAFR knockout mice were placed on either a low-fat (solid) or high-fat (open) diet for 12 weeks. Following dietary treatment, mice were infected with influenza virus followed by bacterial challenge 7 days later. Survival was analyzed by a log-rank (Mantel-Cox) test. *n* = 4 to 5 mice per group. *, *P* < 0.05.

In addition to decreased immune cell populations, we observed increased areas of “active” infection (defined as the presence of intensely influenza virus-positive type II pneumocytes and macrophages and strong staining of alveolar surfaces) in the obese mice at all time points postinfection (Fig. 5a), coupled with enhanced exposure of basement membranes within these sites (Fig. 5b), which may lead to elevated levels of key bacterial receptors and binding sites (11, 31). To test this hypothesis, we measured expression of platelet-activating factor receptor (PAFR), which is associated with increased secondary bacterial infection (32–34) in the lungs of lean and obese mice prior to and 7 days following influenza virus infection. PAFR expression increased ∼10-fold in the lungs of obese mice but not in lean mice (Fig. 5c). To determine the importance of PAFR upregulation, PAFR knockout mice (PAFRKO) were put on control or high-fat diets for 10 weeks, as described elsewhere (35) and then inoculated with influenza virus followed by bacteria. PAFRKO and wild-type mice gained similar amounts of weight on the high-fat diet, and these weight gains were significantly higher than mice that received the control diet (Fig. S5). While severity of secondary bacterial infection was not altered in PAFRKO lean mice, survival and time to death were slightly but significantly improved in the obese PAFRKO mice compared to obese controls (Fig. 5d). Together, these data show that increased bacterial colonization and spread in obese mice are likely due to several mechanisms, including, but not limited to, decreased patrolling innate immune cells that are important for clearance, in addition to increased bacterial binding sites, including the PAFR, a known pneumococcal receptor.

10.1128/mBio.00889-17.5 FIG S5  PAFR knockout mice gain weight similarly to WT mice on a high-fat diet. C57BL/6 (circles) and PAFR knockout (squares) mice were fed control (black) or high-fat (open) diets for 10 weeks. *n* = 4 to 5 animals/group. Download FIG S5, TIF file, 0.3 MB. Copyright © 2017 Karlsson et al. 2017 Karlsson et al. This content is distributed under the terms of the Creative Commons Attribution 4.0 International license.

### Increased mortality is not due to increased bacteremia.

In addition to extensive colonization and outgrowth in the respiratory tract, secondary bacterial infection is also associated with invasive bacteremia into the bloodstream and other extrarespiratory sites in both mice and humans (36, 37). Interestingly, in contrast to the lean animals, blood titers were undetectable in obese animals through 3 days post-bacterial challenge (Fig. S5). Therefore, despite the increased bacterial and viral titers observed in the lungs of obese animals, disease did not progress to the invasive phenotype in these animals. One explanation for this finding is that obese animals may succumb too quickly to allow detection of any invasive disease in this model.

### Obese mice have increased edema.

**FIG 6:**
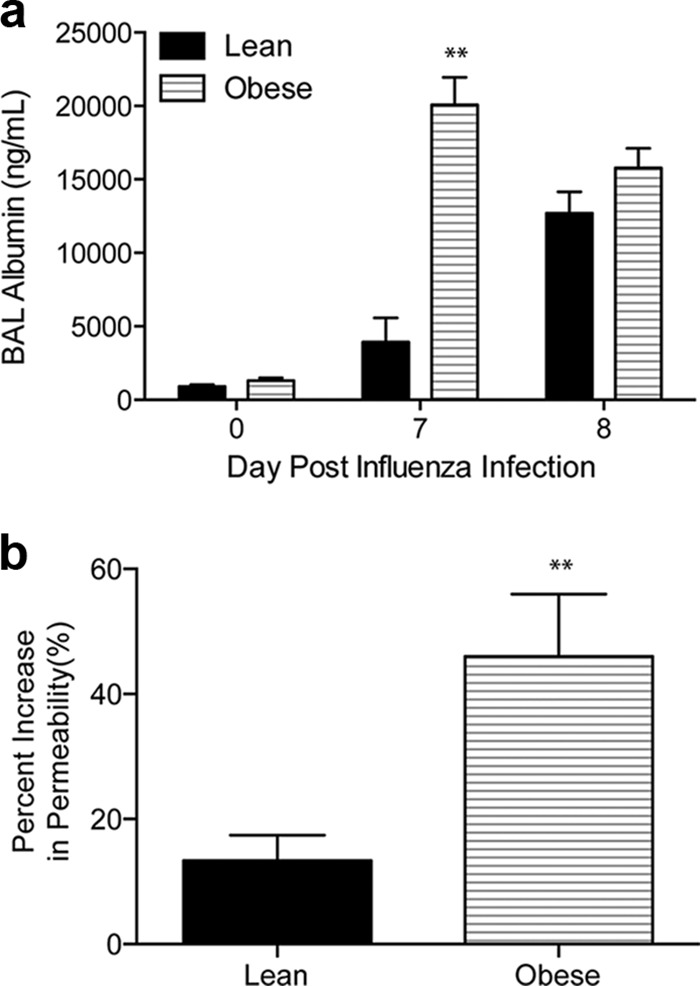
Obese mice have increased lung permeability prior to secondary bacterial challenge. (a) Albumin levels were measured in bronchoalveolar lavage fluid by ELISA. (b) Lung permeability was assessed in an assay using Evan’s blue dye prior to and 7 days post-influenza virus infection. Data are representative of two separate experiments with 5 mice/group/time point. *, *P* < 0.05; **, *P* < 0.005.

Regardless of the mechanism(s) underlying the increased viral and bacterial titers, we hypothesized that the extensive microbial spread and ensuing damage result in increased lung permeability and edema in coinfected obese mice, which could ultimately lead to impaired lung function and mortality (60). Indeed, obese mice had higher levels of albumin in bronchioalveolar lavage fluid (BALF), indicating disruption of the pulmonary epithelium and vascular endothelium (Fig. 6a), as well as increased lung permeability, as quantified by injecting lungs with Evans blue dye and measuring the amount of dye in the lungs 1 day post-bacterial challenge, compared to lean controls. Obese mice had an ∼50% increase in lung permeability, compared to an ∼15% increase in lean controls (Fig. 6b), highlighting an immediate and significant increase in lung permeability in obese mice, which could be the ultimate cause of mortality due to a possible decrease in lung functionality.

### Vaccinations are not protective against secondary bacterial infection in obese mice.

**FIG 7:**
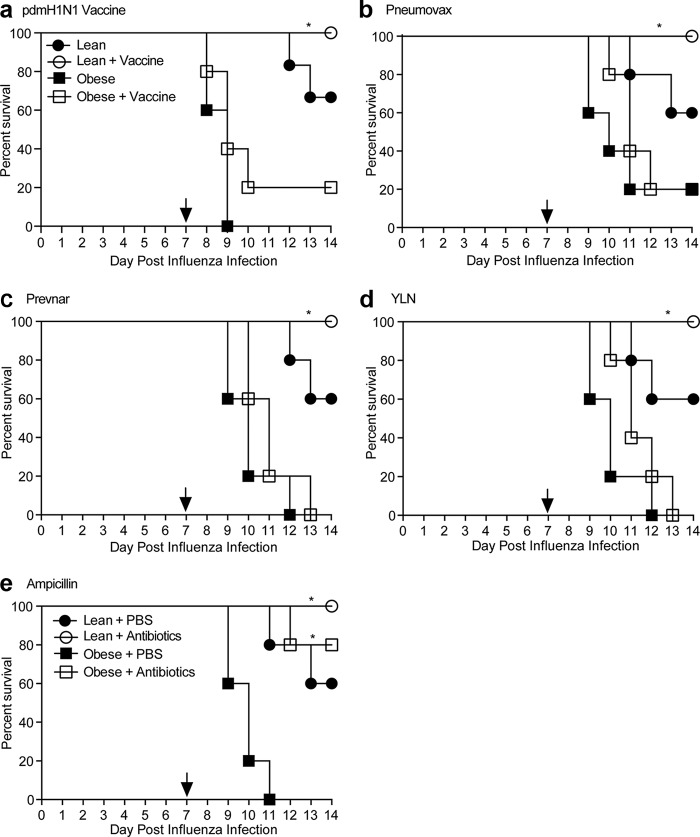
Antibiotic therapy, but not influenza or pneumococcal vaccination, is protective against secondary bacterial infection-associated mortality in obese mice. Lean or obese mice were vaccinated with PBS (solid symbols) or influenza virus vaccine (a), pneumococcal polysaccharide vaccine (b), pneumococcal conjugate vaccine (c), or protein-based pneumococcal vaccine (d). Following the boost, mice were infected with influenza virus followed by bacterial challenge 7 days post-influenza virus infection. (e) Mice were treated with ampicillin for 3 days post-bacterial challenge. Mortality was monitored for 7 days following bacterial challenge. Survival was analyzed by a log rank (Mantel-Cox) test. *n* = 5 mice/group. *, *P* < 0.05.

Obese mice are more likely to develop severe disease if exposed to bacteria post-influenza virus infection. The major question is how do we protect this population? The Centers for Disease Control and Prevention (CDC) recommends that people receive the influenza vaccine to prevent community-acquired pneumonia, including pneumonia caused by secondary bacterial infection (38, 39). Additionally, polysaccharide conjugate vaccines have been shown to reduce the severity of pneumonia in high-risk populations (40); however, studies of effectiveness of bacterial vaccination in mice are inconclusive (41), and success rates of influenza vaccines are significantly reduced in obese mice (42). In addition, capsule-based vaccine strategies may not be effective against all pneumococcal serotypes (43); further studies are warranted that utilize novel protein-based vaccines (44). Thus, we tested the efficacy of influenza and three pneumococcal (two capsule-based and one protein-based) vaccines in protecting against severe disease in coinfected lean and obese mice. Influenza vaccination resulted in complete protection of lean mice but failed to protect obese mice (Fig. 7a). Similar to previous results (42) and likely due to a lack of seroconversion (Fig. S6a), only 20% of the vaccinated obese mice survived coinfection, compared to 100% survival of lean animals. Results were even more discouraging with the all pneumococcal vaccines, with no differences in survival in the obese animals, in contrast to complete protection in lean mice (Fig. 7b). Again, these results may be the result of decreased seroconversion following vaccination (Fig. S6b).

10.1128/mBio.00889-17.6 FIG S6  Obese mice do not develop invasive bacteremia following secondary bacterial challenge. Lean and obese mice were infected with influenza virus A/California/04/2009 and then challenged with *S. pneumoniae* strain D39x at day 7 post-influenza virus infection. Blood bacterial titers were monitored prior to and every 24 h post-bacterial challenge for 72 h. Data are representative of three separate experiments. *n =* 3 mice/group/time point. *, *P* < 0.05. Download FIG S6, TIF file, 0.3 MB. Copyright © 2017 Karlsson et al. 2017 Karlsson et al. This content is distributed under the terms of the Creative Commons Attribution 4.0 International license.

### Antibiotic treatment decreases mortality from secondary bacterial infection in obese mice.

Lack of vaccine protection in obese mice necessitates further studies on possible prophylactic and therapeutic strategies. Previous studies have shown antibiotic agents to be efficacious in preventing secondary bacterial morbidity and mortality in mouse model (45). Since their discovery (46), beta-lactam agents have been the recommended treatment for patients with pneumococcal pneumonia and as a primary therapy for bacterial pneumonia following influenza virus infection (47). Data on antibiotic efficacy in obese patients are limited, and data suggest that obesity is associated with altered antibiotic pharmacokinetics and pharmacodynamics (48, 49). Therefore, we decided to test whether beta-lactam treatment, specifically ampicillin, improved mortality from secondary bacterial infection in obese mice. Encouragingly, treatment of obese mice with ampicillin following secondary bacterial challenge resulted in an 80% increase in survival (20% mortality versus 100%) compared to vehicle-treated controls (Fig. 7e). These results suggest that antibiotic treatment could serve as an adequate countermeasure to bacterial infections in the influenza virus-infected obese host, and further work is warranted on these protective measures.

## DISCUSSION

In these studies, we demonstrated that secondary bacterial infections result in higher mortality in influenza virus-infected obese mice compared to lean animals, regardless of the viral or bacterial strain or the time of administration. The finding that mortality is exacerbated in obese mice infected with human seasonal, pandemic, and even influenza virus B strains is concerning. As influenza viruses become more adapted to mammalian hosts, increased glycosylation of surface proteins, lowered neuraminidase activity, and mutated or nonfunctional PB1-F2 proteins are thought to lower disease-associated phenotypes and decrease the efficiency of priming for bacterial infections (15); however, this does not appear to be the case in the obese host. This finding is particularly important for an increasingly obese world. Limited data are available on the exact etiology of pneumonia in the obese population. Several studies have shown obese individuals to be at increased risk for pneumonia while, paradoxically, others have shown negative associations between bacterial infection and pneumonia in obese populations (65, 66). Therefore, further epidemiological studies are needed to specifically determine the severity of secondary bacterial infections in obese humans following influenza virus infection. In addition, further work will focus on specific metabolic changes associated with obesity as they pertain to influenza and secondary bacterial infection in the obese host.

The increased disease severity in obese mice appears to stem from a “perfect storm” of events that lead to viral and bacterial outgrowth following bacterial infection. The elevated viral load and spread, as indicated by more areas of persistent active infection, lead to increased exposure of bacterial receptors, such as PAFR, that facilitate host invasion. Alterations in key innate immune cells could also contribute to the amplified bacterial colonization and ensuing lung injury. However, other mechanisms warrant investigation. For example, why do bacterial titers increase exponentially in obese mice? As described, differences in alveolar macrophages observed between lean and obese hosts could be associated with altered bacterial clearance (24); however, other adaptive immune mechanisms could also be associated with an altered response to secondary bacterial challenge. Natural killer (NK) cells, T regulatory (T_reg_) cells, and T helper cells that produce interleukin-17 (referred to as type 17 immunity here) have all been shown to be important for resolution of bacterial infection following influenza virus insult (29, 30). Obesity has been shown to alter both NK cell cytotoxicity (4) and T_reg_-suppressive responses (50) during influenza virus infection which could negatively affect bacterial clearance. Further work needs to be done on the contribution of these cell types as well as type 17 immunity in the obese host in the context of secondary bacterial infection.

Another major question is why do viral titers increase during coinfection? Adequate innate antiviral responses as well as functional adaptive mechanisms are required to effectively clear influenza virus infection. Obesity has been shown to negatively impact both innate and adaptive responses to primary influenza virus infection (1, 8, 13). Both type I and type II antiviral interferons (IFNs), as well as influenza virus-specific CD8^+^ T cell responses, are involved in viral clearance as well as the response to bacterial superinfection (28, 30). Obesity has been shown to alter IFN-α and IFN-β expression during primary influenza virus infection (4) as well as influenza virus-specific CD8^+^ T cell effector function and memory (35). Therefore, these mechanisms, coupled with the obese immune system attempting to respond to secondary bacterial challenge, could tip the scales toward loss of control of viral replication. Further studies will focus on the contribution of these antiviral defenses in the context of secondary bacterial infection in the obese host.

Of great concern is the inability of either influenza or pneumococcal vaccines to protect obese mice from severe secondary bacterial infection. The CDC recommends vaccination against influenza as a preventative for community-acquired pneumonia (39); however, previous studies in our lab and others have demonstrated that influenza vaccination does not appear to be protective against influenza virus infection in obese mice and humans. This lack of vaccine-associated defense occurs even when adjuvants are used to boost the serological response above theoretically protective levels in obese mice (42, 51–53). In addition, there are limited data on the efficacy of antibacterial vaccination against secondary pneumococcal pneumonia in mice (41), and no studies to date have utilized these vaccines in an obese host. Similar to influenza vaccination, bacterial vaccinations that resulted in decreased seroconversion were unable to protect obese animals from severe secondary bacterial infection. Encouragingly, in contrast to vaccination, administration of antibiotics was able to mitigate mortality in obese mice. Further studies are necessary to understand this lack of protection from vaccination and to further investigate mechanisms of prophylactic and therapeutic strategies by using antivirals and antibiotics in this expanding population.

Taken together, these studies indicate an urgent need to understand secondary bacterial pneumonia in the obese host. The obese host appears to have a higher preset state of inflammation and expression of bacterial receptors in the lung, and these likely accelerate and exaggerate bacterial and viral virulence when presented as a coinfection. The relatively modest efficacy of systemic immune responses to vaccines in controlling pneumonia appears to be greatly overmatched by the course of coinfection in the obese host. The global epidemic of obesity coupled with the increased severity of influenza virus infection in obese mice and humans, as well as decreased prophylactic and therapeutic options, make it imperative to further understand complications of respiratory infections in this expanding population.

## MATERIALS AND METHODS

### Ethics statement.

All experimental procedures were approved by the St. Jude Children’s Research Hospital Institutional Biosafety Committee (IBC) and Animal Care and Use Committee (ACUC) and were conducted in compliance with the *Guide for the Care and Use of Laboratory Animals* (67). These guidelines were established by the Institute of Laboratory Animal Resources and approved by the Governing Board of the U.S. National Research Council. All experiments were performed in a biosafety level 2 facility accredited by the American Association of Laboratory Animal Science.

### Viruses, purification, and determination of titers.

All viruses [A/Puerto Rico/8/1934, A/California/04/2009, A/California/04/2009-PA-NanoLuciferase (NLuc), A/Victoria/361/2011, A/Brisbane/59/2007, A/Indiana/08/2011, and B/Wisconsin/1/2010] were propagated in the allantoic cavity of 10-day-old specific-pathogen-free embryonated chicken eggs at 37°C. Allantoic fluid was harvested, cleared by centrifugation, and stored at −80°C as described previously (54, 55). Viral titers were determined by both luminescent and standard 50% tissue culture infective dose (TCID_50_) analysis as previously described (22, 54). A/California/04/2009 virus was purified as described previously (56). Briefly, virus preparations were overlaid onto a 30 to 60% discontinuous sucrose gradient and then centrifuged for 90 min at 26,000 rpm. The virus layer was extracted, pelleted by another round of centrifugation for 60 min, and then resuspended in phosphate-buffered saline (PBS).

### Bacterial strains and titers.

*Streptococcus pneumoniae* was grown on plates of tryptic soy agar (TSA; EMD Chemicals, New Jersey) supplemented with 3% sheep blood or in C+Y, a defined semisynthetic casein liquid medium (57) supplemented with 0.5% yeast extract. *S. pneumoniae* strain D39x (serotype 2) was transformed with the lux operon (Xenogen) to induce bioluminescence (21).

Cultures of *S. pneumoniae* were inoculated from either frozen stock or newly streaked TSA blood plates and incubated at 37°C in 5% CO_2_. For bacterial burden and survival studies, strains were grown in C+Y medium to an optical density at 620 nm (OD_620_) of 0.4 and diluted according to a previously determined standard curve. Bacteria were enumerated to ensure that the proper amounts of bacteria were used in infections.

### Cells.

Madin-Darby canine kidney (MDCK) cells were cultured in Eagle’s minimum essential medium (MEM; Mediatech, Manassas, VA) supplemented with 2 mM glutamine and 10% fetal bovine serum (FBS; Gemini BioProducts, West Sacramento, CA) and grown at 37°C under 5% CO_2_.

### Animal experiments. (i) Generation of DIO mice.

Weanling, male C57BL/6J mice (Jackson Laboratories, Bar Harbor, ME) or PAFR knockout mice (58) were fed either a low-fat or high-fat diet *ad libitum* (Research Diets, New Brunswick, NJ, USA) for 12 to 16 weeks as described previously (35).

### (ii) Infection experiments.

Coinfection experiments were performed as described previously (14). Briefly, 8-week-old C57BL/6 (lean) and B6.Cg-*Lepob*/J (*ob*/*ob*) mice (Jackson Laboratory, Bar Harbor, ME) along with diet-induced lean and obese mice were anesthetized with 2.5% inhaled isoflurane (Baxter, Deerfield, IL) and intranasally inoculated with sublethal doses of influenza virus in a total volume of 30 µl. Three, 7, or 10 days after influenza virus infection, mice were anesthetized with 2.5% inhaled isoflurane and intranasally inoculated with 100 CFU of *S. pneumoniae*, and then monitored daily for clinical signs of infection and weighed every 24 hours postinfection (59). Moribund mice that had lost more than 30% body weight were humanely euthanized. At days 7, 8, 9, and 10 postinfection, mice were euthanized by CO_2_ asphyxiation, and tissues (lung, bronchoalveolar lavage fluid, nasal wash, blood) were harvested and processed immediately or stored at −80°C for future analysis.

### (iii) Vaccination.

Five- to six-week-old C57BL/6 and B6.Cg-*Lepob*/J mice were bled for baseline serum samples, lightly anesthetized with isofluorane, and then vaccinated intramuscularly (*n* = 5/group) with PBS, 4 hemagglutinating units of β-propriolactone-inactivated, sucrose-purified A/California/04/2009 virus, Pneumovax (Merck), Prevnar (Wyeth), or protein-based YLN vaccine (44). Prevnar and YLN vaccine were supplemented with alum as an adjuvant. Three weeks following initial vaccination, mice were then boosted with a second dose of vaccine or PBS. Two weeks after the boost, mice were bled for serum collection and then challenged as described above. Serological assessments for influenza virus (42) and bacterial vaccination (44) were performed as described previously.

### (iv) Antibiotic treatment.

Antibiotic treatments were performed as described previously (45). Briefly, mice were given ampicillin (50 mg/kg/day; Sigma-Aldrich Co., St. Louis, MO) or PBS vehicle intraperitoneally twice daily in divided doses (50 mg/kg/day) for 3 days post-bacterial infection.

### (v) Pathology.

At 0, 7, 8, and 9 days post-influenza virus infection, deeply anesthetized mice were perfused with 10% neutral buffered formalin, and tissues were collected and processed for hematoxylin and eosin staining and immunohistochemistry for NP staining by the St. Jude Children’s Research Hospital Veterinary Pathology Core (VPC) Facility as described elsewhere (60). Slides were analyzed for influenza virus spread and inflammation via blinded scoring by the VPC.

### (vi) Imaging.

For bioluminescent viral challenge, mice were euthanized and then imaged for 3 min using an IVIS charge-coupled-device (CCD) camera (22, 61). For pneumococcal challenge, mice were imaged for 60 s using an IVIS CCD camera (Caliper Life Sciences, Alameda, CA) daily to monitor *in vivo* pneumococcal pneumonia development. Total photon emission from selected and defined areas within the images of each mouse was quantified using LivingImage software (Caliper Life Sciences) as described previously (60, 62).

### (vii) Flow cytometry.

BALF was collected on days 0, 7, and 8 post-influenza virus infection by flushing the lungs twice with 1 ml PBS. BALF was centrifuged, and the cell pellet was used for cell analysis. Cells were treated with Fc block (BD Biosciences) and surface stained with the following antibodies (at 1 µg/ml): anti-CD11b (clone M1/70, BioLegend), anti-CD11c (clone HL3; BD Biosciences), anti-F4/80 (clone BM8; BioLegend), and anti-Ly6G (clone 1A8; BioLegend). Data were acquired for equivalent numbers of cells on a FACSCanto II flow cytometer (BD Biosciences) and analyzed with FlowJo software version 9. Cell types were defined as previously described (24). Briefly, neutrophils were defined as CD11b^hi^ Ly6G^hi^ cells, and alveolar macrophages were defined as CD11c^hi^ F4/80^hi^ CD11b^−^ cells.

### Lung permeability assays. (i) Evans blue permeability assay.

*In vivo* lung permeability was assessed as described elsewhere (60). Briefly, at day 7 post-influenza virus infection, mice were administered 20 mg/kg Evans blue dye (Sigma) by retro-orbital injection. The dye was allowed to circulate for 2 h before blood was collected for serum samples, and then mice were perfused with 30 ml PBS and sacrificed. Lungs were harvested and incubated in formamide (Sigma) at 65°C. After 48 h, lungs were removed from formamide, dried at 60°C for an additional 72 h, and then weighed. Evans blue dye was measured in lung and serum by spectrophotometry at 620 nm. The lung permeability index was calculated using the following formula: [(lung absorbance/serum absorbance) × dilution factor]/lung dry weight (in grams) (63).

### (ii) Albumin ELISA.

BALF and albumin concentrations were measured by enzyme-linked immunosorbent assay (ELISA; Bethyl, Montgomery, TX) according to the manufacturer’s instructions (60).

### Statistical analyses.

Statistical analyses were performed using JMP Statistical Software (SAS Institute, Cary, NC) and GraphPad Prism (GraphPad Software, Inc., La Jolla, CA). Survival comparisons were analyzed by a log rank (Mantel-Cox) test. Nonparametric data were analyzed using the Kruskal-Wallis test (α = 0.05), and normally distributed data were analyzed by a two-way analysis of variance with host group and time postinfection as the main effects. Student’s *t* test was used for *post hoc* comparisons between host groups, and Tukey’s honestly significant test was used for *post hoc* comparisons among the data based on time postinfection. Differences were considered significant at a *P* level of <0.05.

10.1128/mBio.00889-17.7 FIG S7  Vaccination against influenza virus or pneumococcus results in reduced seroconversion in obese mice. Lean (solid) and obese (open) mice were vaccinated with (a) influenza vaccine or (b) pneumococcal vaccines. (a) Postboost serum was analyzed for hemagglutination inhibition (HAI) and microneutralization (MN) against influenza virus A/California/04/2009 (pdmH1N1). *n* = 5 mice/group. (b) Postboost serum was analyzed for protein-based pneumococcal vaccine (YLN; circles), pneumococcal conjugate vaccine (Prevnar; squares), or adjuvant alone (alum; triangles) by determining anti-YLN and anti-TIGR4 IgG titers. Titer data are presented as individual data points plus means. *n* = 10 mice/group. *, *P* < 0.05. Download FIG S7, TIF file, 0.6 MB. Copyright © 2017 Karlsson et al. 2017 Karlsson et al. This content is distributed under the terms of the Creative Commons Attribution 4.0 International license.
