# Supplementary figures and images for: A Perfect Storm: Increased Colonization and Failure of Vaccination Leads to Severe Secondary Bacterial Infection in Influenza Virus-Infected Obese Mice
Source: mBio. 2017 Sep 19;8(5):e00889-17. doi: 10.1128/mBio.00889-17 (PMC5605935; doi:10.1128/mBio.00889-17)

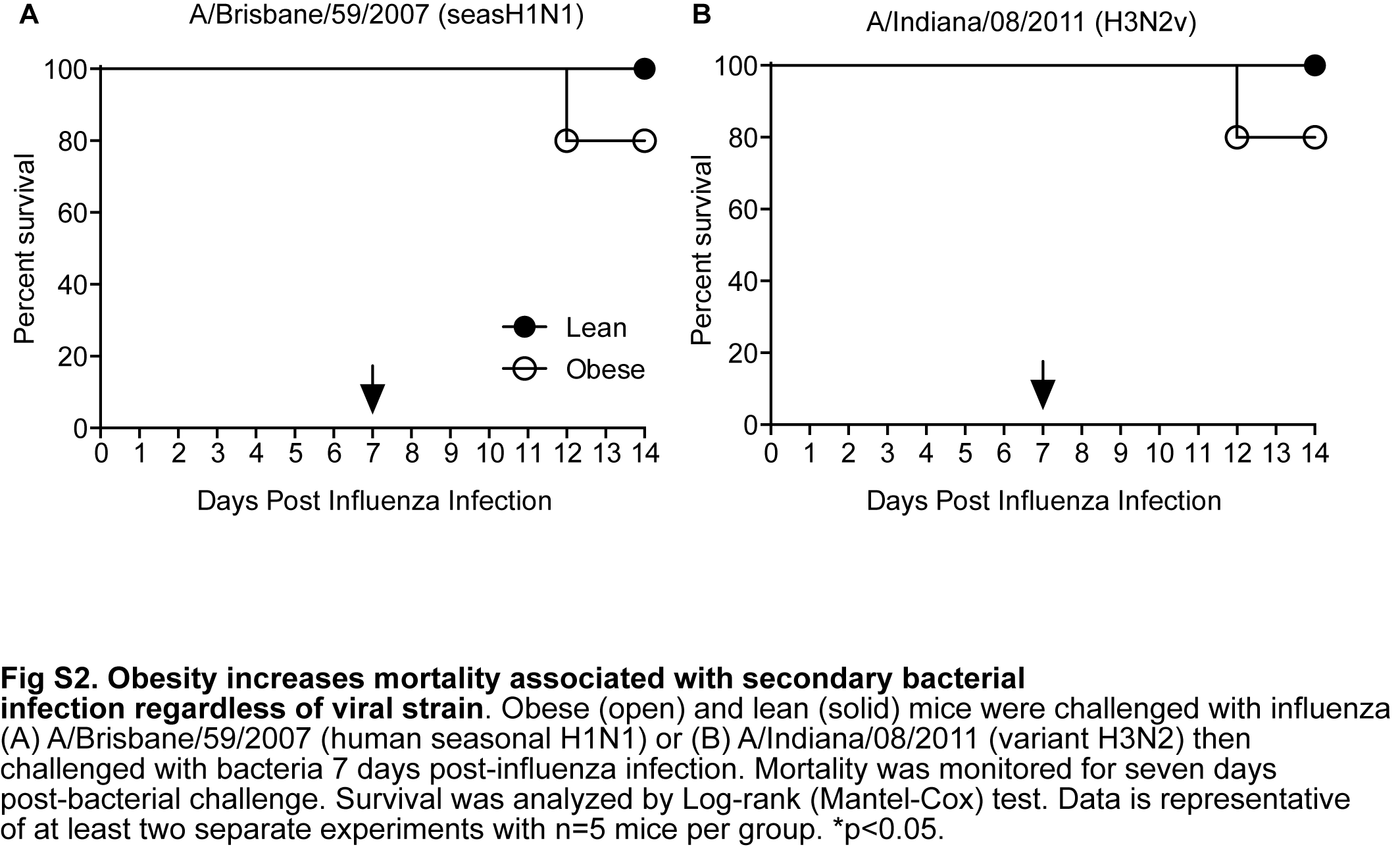

Supplement: FIG S2 [file mbo004173491sf2.tif]

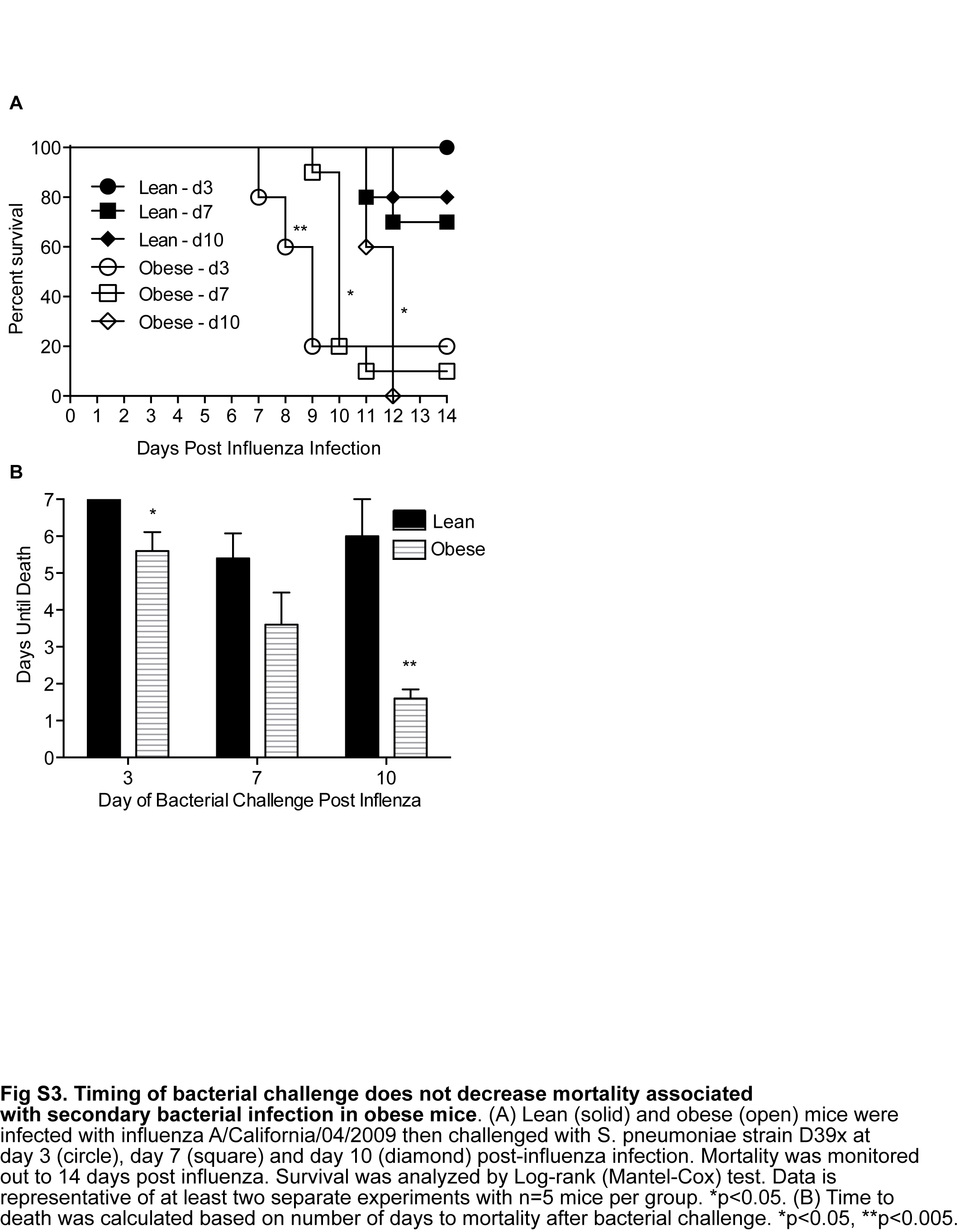

Supplement: FIG S3 [file mbo004173491sf3.tif]

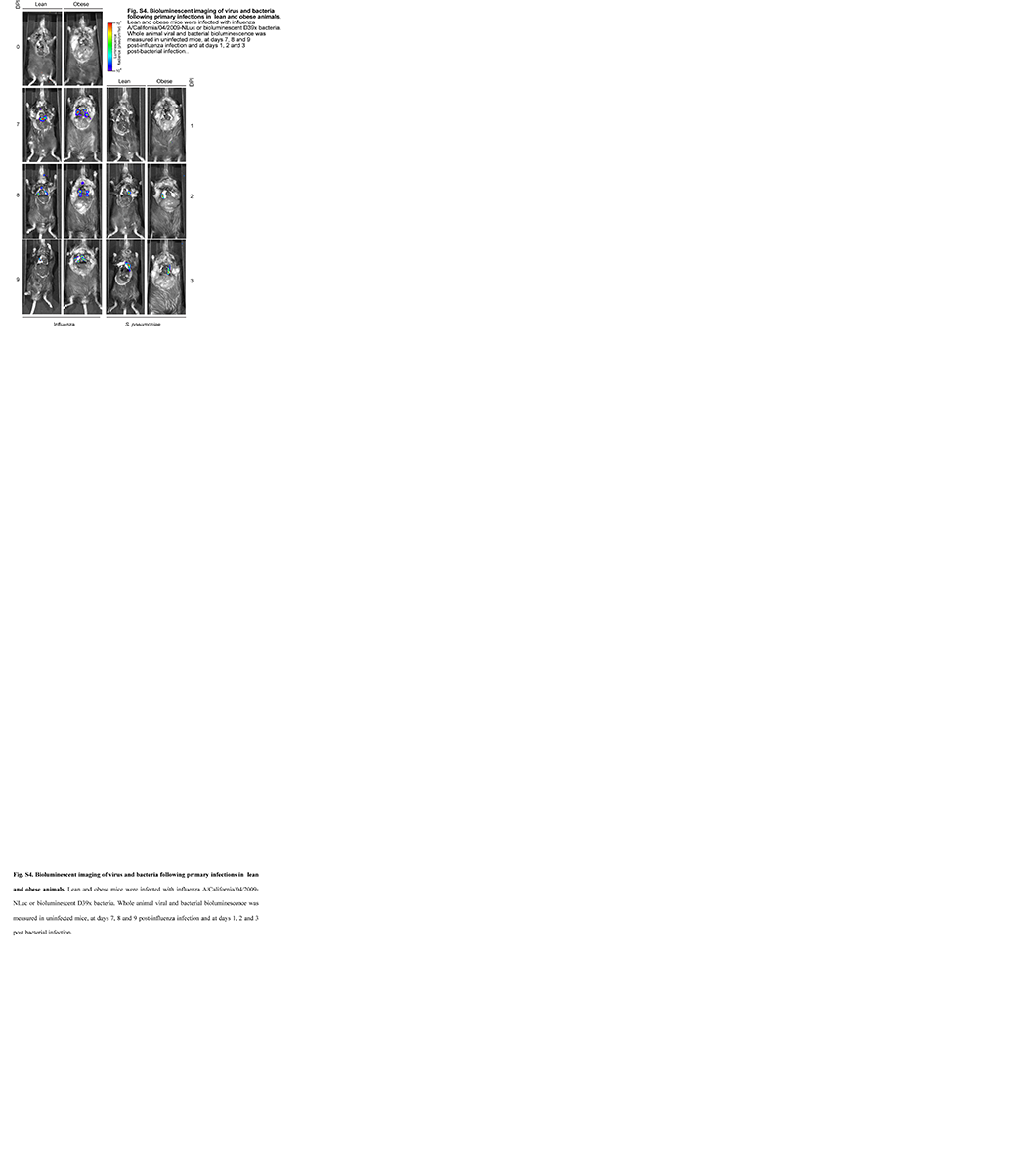

Supplement: FIG S4 [file mbo004173491sf4.tif]

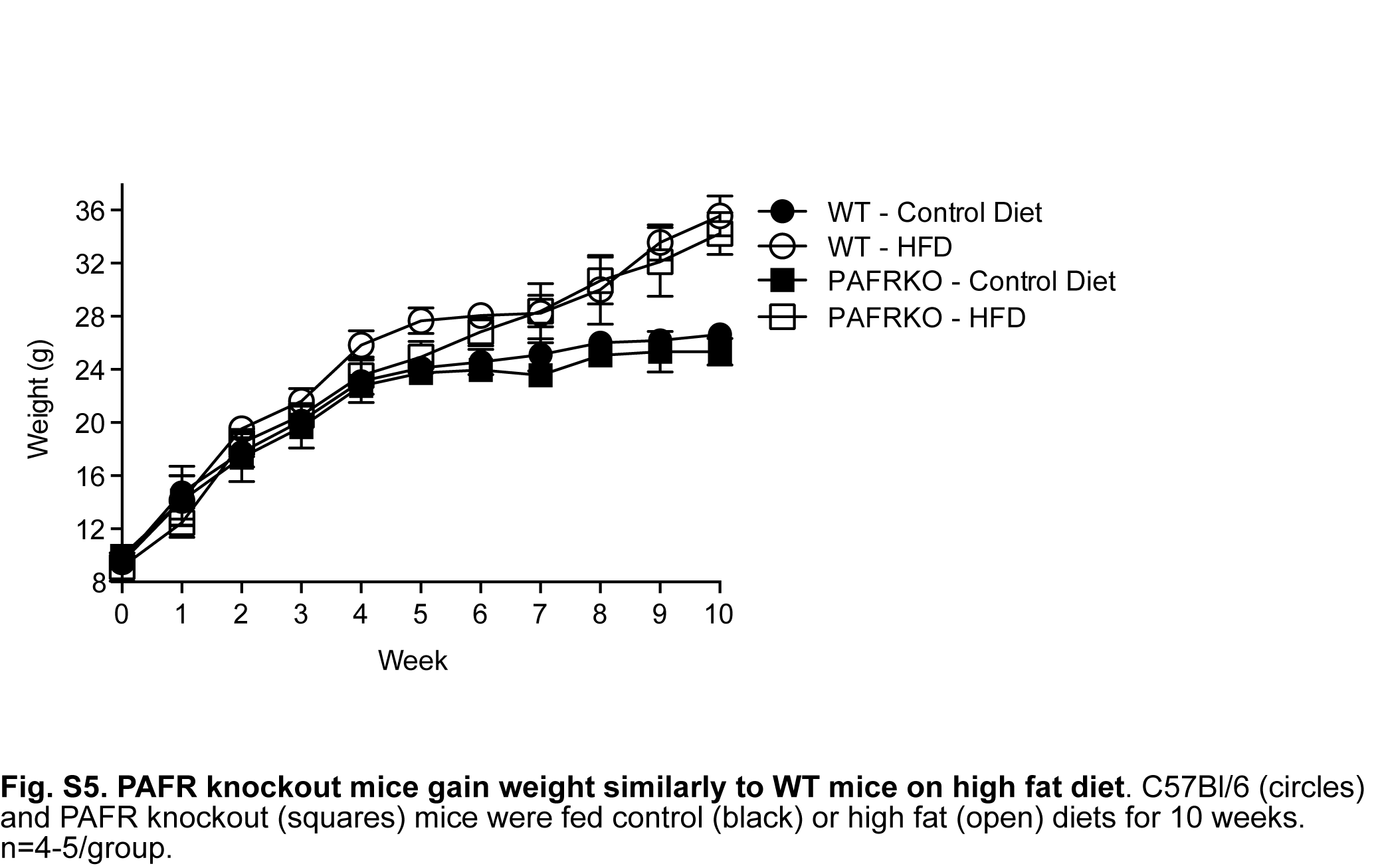

Supplement: FIG S5 [file mbo004173491sf5.tif]

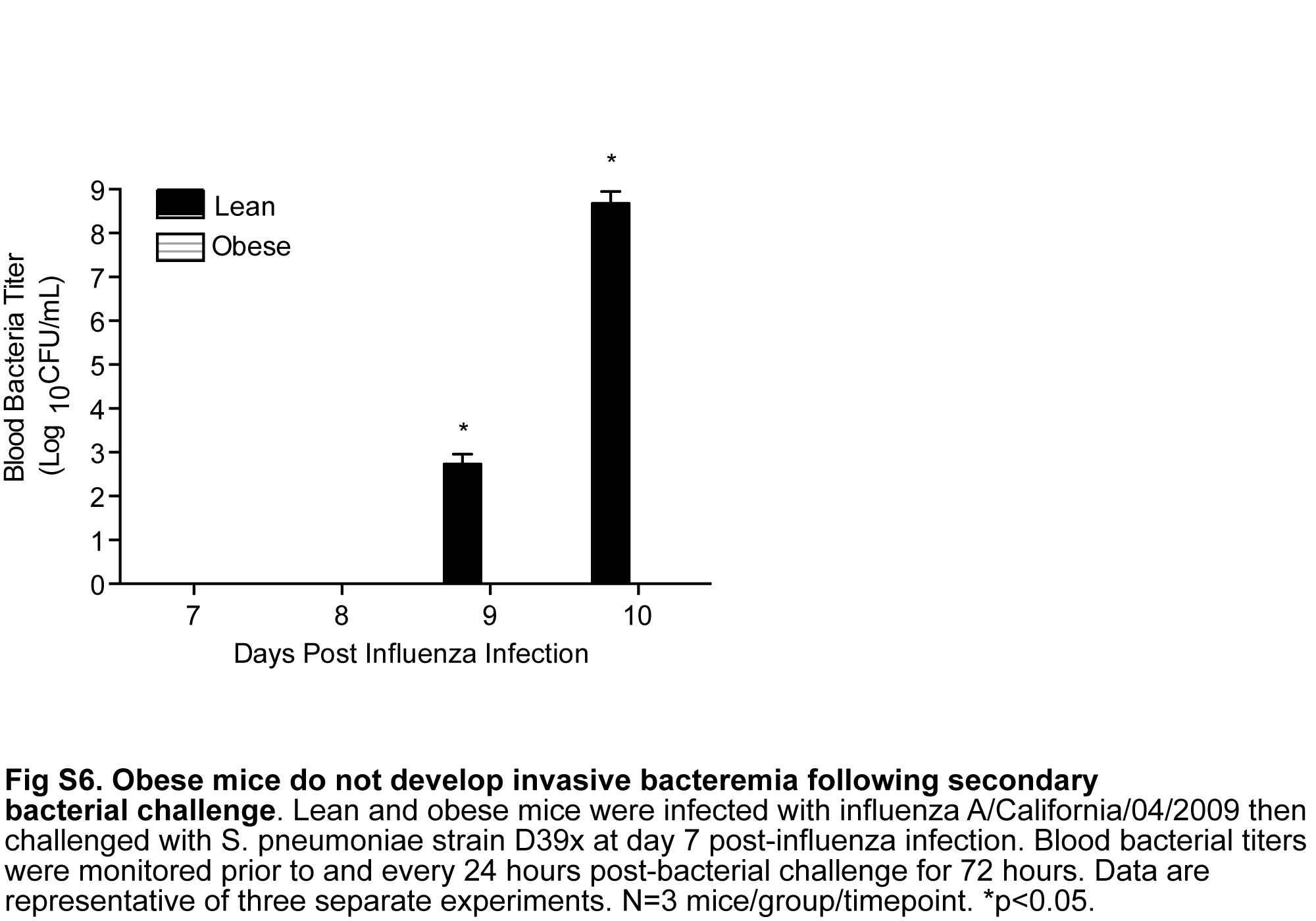

Supplement: FIG S6 [file mbo004173491sf6.tif]

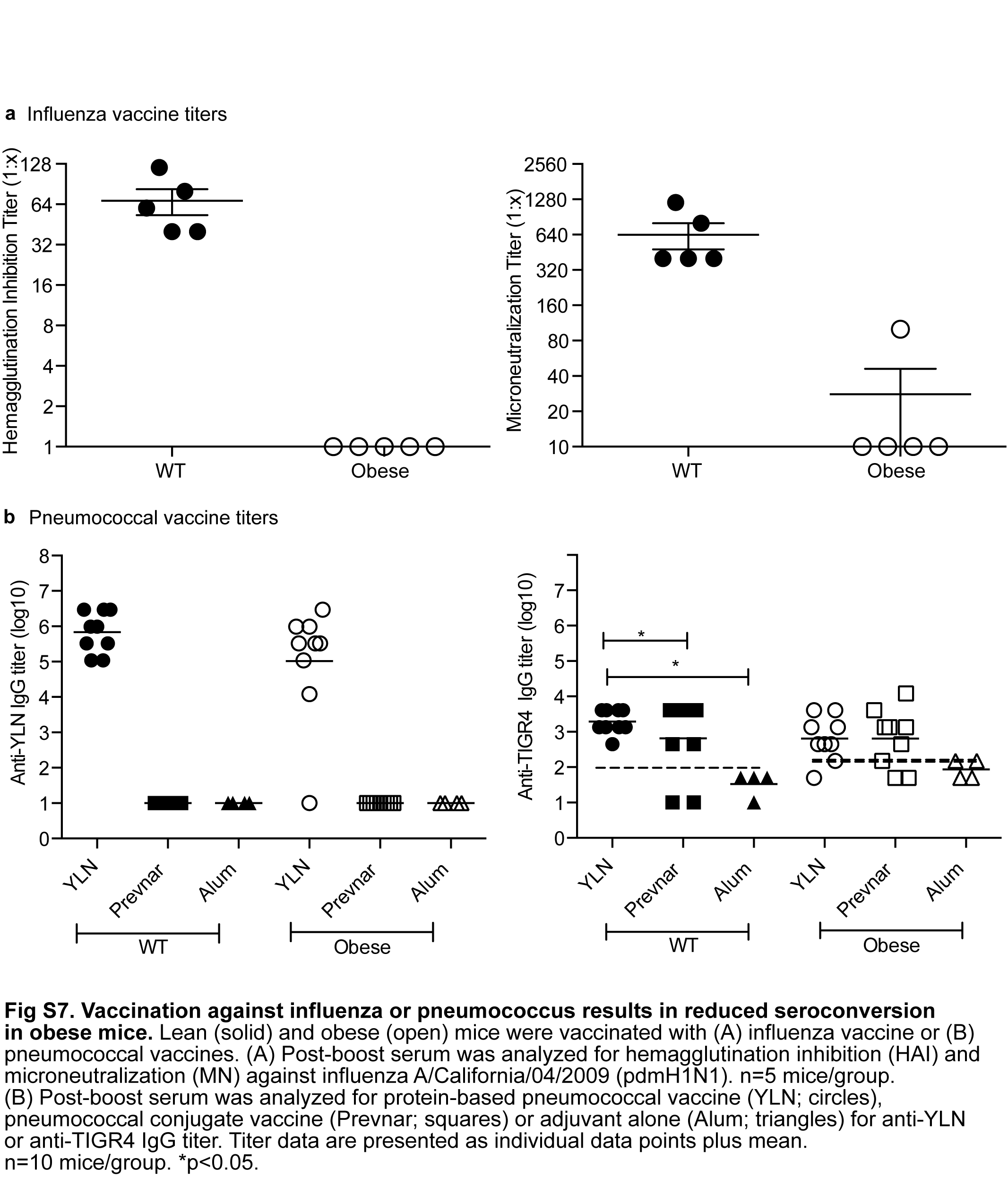

Supplement: FIG S7 [file mbo004173491sf7.tif]
